# Optimum Design Rules for CMOS Hall Sensors

**DOI:** 10.3390/s17040765

**Published:** 2017-04-04

**Authors:** Marco Crescentini, Michele Biondi, Aldo Romani, Marco Tartagni, Enrico Sangiorgi

**Affiliations:** 1 Department of Electrical, Electronic and Information Engineering “G. Marconi”—DEI, University of Bologna, Cesena Campus, Via Venezia 52, 47521 Cesena, Italy; aldo.romani@unibo.it (A.R.); marco.tartagni@unibo.it (M.T.); enrico.sangiorgi@unibo.it (E.S.); 2 Advanced Research Center on Electronic Systems (ARCES), University of Bologna, Cesena Campus, Via Venezia 52, 47521 Cesena, Italy; bionds86@alice.it

**Keywords:** Hall sensors, current-related sensitivity, power consumption, design rules, 3D physical simulations, Hall effect sensor design

## Abstract

This manuscript analyzes the effects of design parameters, such as aspect ratio, doping concentration and bias, on the performance of a general CMOS Hall sensor, with insight on current-related sensitivity, power consumption, and bandwidth. The article focuses on rectangular-shaped Hall probes since this is the most general geometry leading to shape-independent results. The devices are analyzed by means of 3D-TCAD simulations embedding galvanomagnetic transport model, which takes into account the Lorentz force acting on carriers due to a magnetic field. Simulation results define a set of trade-offs and design rules that can be used by electronic designers to conceive their own Hall probes.

## 1. Introduction

Magnetic field sensors are valuable for many kinds of applications: from industrial to automotive (e.g., position sensors), from home applications (e.g., current sensors) to smartphones (e.g., compass). Among magnetic sensors, the Complementary Metal-Oxide-Semiconductor (CMOS) Hall sensor has a primary role thanks to its compatibility with standard microelectronic processes. A silicon-based Hall probe shows worse performance than a discrete Hall probe implemented with high-mobility compound semiconductors such as GaAs or InSb [1]. However, the possibility of integrating magnetic sensors and signal conditioning circuits into the same substrate allows efficient implementation of several circuit techniques improving Hall performance, such as offset reduction, temperature stabilization and nonlinearity correction [2,3,4]. Given the pervasive use of CMOS Hall sensors, it will be useful to have a set of simple rules to customize the sensor design to the target application.

A macroscopic description of the Hall effect offers a useful theory, though with poor physical insights. On the contrary, quantum theory provides a deep understanding of the processes acting on each single carrier, albeit with an awkward mathematical description unsuitable for sensor design. In this context, numerical simulations of magnetic devices are a promising approach. Although the first research articles about technology-computer-aided-design (TCAD) appeared in the 1990s [5,6], commercial TCAD software coping with galvanomagnetic effects—that is, the full implementation of the Lorentz force—has only recently been made available [7].

The majority of the literature about Hall sensor presents a single and novel Hall device with particular characteristics [8,9,10] or a novel and better-performing sensing system based on a specific Hall probe [11,12,13,14,15,16]. There are a few articles investigating the performance of general Hall probes focusing on the sole offset voltage [17,18] or comparing a limited set of given geometries [19,20,21] without providing general rules. Jankovic et al. [18] study the performance of well-defined cross-shaped and vertical Hall sensors so as to define an electrical equivalent SPICE model. The paper is limited to the analysis of the realized sensors without discussing the effects of geometries, bias or other design parameters on the final performance. Paun et al. [20] make use of three-dimensional (3D) TCAD simulations to compare among five different shapes of Hall sensors. The paper mainly analyzes and compares the sensitivities and the offsets so as to define the best sensor shape. However, no discussion on the exact values of length, width, doping or any other design parameter is made; hence, no rules for designers are provided.

This manuscript exploits advanced 3D TCAD tools to study the behavior of general Hall probes, irrespective of the chosen geometry. It focuses on the effects of design parameters, such as the aspect ratio or the positions of the contacts, on the final performance of the Hall probe, like sensitivity, power consumption, and bandwidth. The final scope of the article is providing a set of simple rules for designers so they can choose the best geometry, length, and bias for the target application. Offset and temperature are not covered by this manuscript as there is already a significant amount of literature about those effects [3,17,19,22,23,24]. This manuscript is organized as follows: Section 2 describes the modeled sensors, the basic theory of the Hall effect and the numerical simulator. Section 3 presents all the simulation results and compares some of them with measurements, while Section 4 provides an overall discussion and defines trade-offs and design rules.

## 2. Materials and Methods

### 2.1. Devices

**Figure 1:**
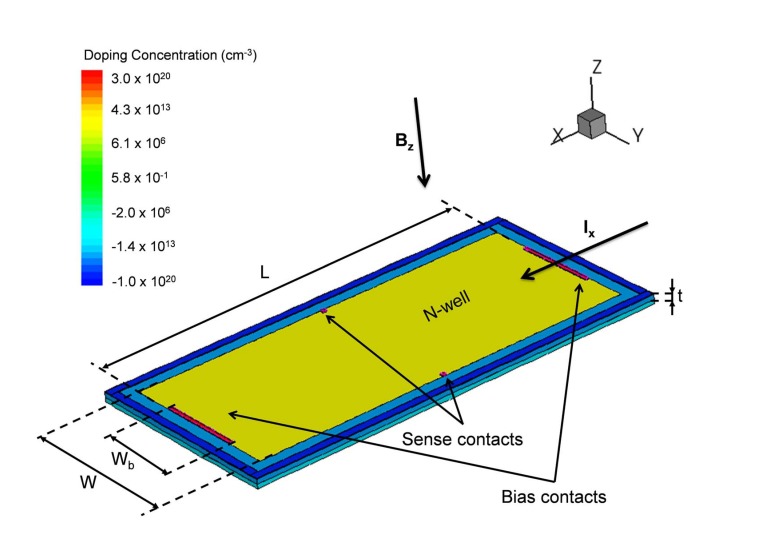

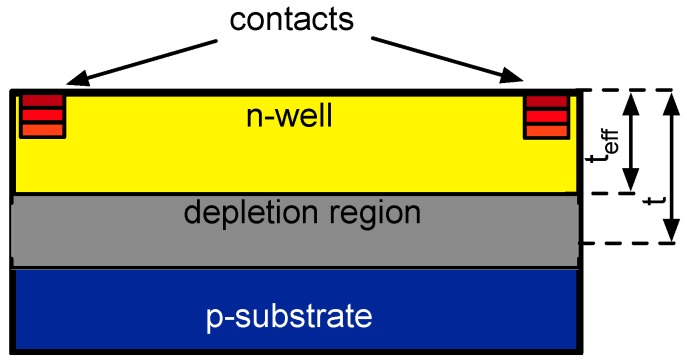
Three-dimensional (3D) model and cross-section of the Hall sensor.

The simulated Hall probes are composed of lightly doped n-well (yellow region in Figure 1) with constant concentration of donor atoms ($N_{D}=2\;\times \;10^{16}{\;\mathrm{cm}}^{-3}$) and four highly doped contacts placed close to the edges of the n-well. The n-well has a rectangular shape so as to get general results regardless of the actual geometry. Width W and length L of the n-well are swept from 20 µm to 140 µm, which are typical sizes. Two contacts facing each other are used to bias the sensor (named bias contacts) and the other two contacts are used to sense the Hall voltage (named sense contacts). Commonly, bias contacts are distinct from sense contacts. In the following simulations, bias contacts are 1 µm long and *W_b_* = *W*/2 wide, unless otherwise stated; sense contacts are placed at the middle of the x-axis, that is the bias-current axis, close to the edges along the y-axis, and are small square contacts 1 µm wide.

All the contacts are 0.4 µm thick and they are modeled by three vertical layers (z-axis) with lower doping concentration going deep into the substrate. This is a simple model for a low-energy ion implantation with projected range close to the surface. A 5 µm thick p-type epitaxial layer surrounds the n-well by 5 µm along all directions. The voltage of the p-type layer is set to ground by long contacts all around the probe (dark blue in Figure 1). This is a very general structure that can be improved by adopting modern technological solutions, such as the creation of a p-layer above the sensitive n-well for sensitivity enhancement [13,15].

### 2.2. Theory

The macroscopic description of the Hall effect is summarized hereafter. When a magnetic field is applied orthogonally to an electric current that is flowing through a material, thus the flow of electrons bends from the original path and a small voltage, named Hall voltage *V_H_*, arises orthogonally to both current and magnetic field. The Hall voltage is given by:

(1) $$V_{H}=S_{I}\cdot I_{x}\cdot B_{z},$$

where *S_I_* is the current-related sensitivity, *I_x_* is the component of the bias current along the x-axis and *B_z_* is the component of the magnetic field along the z-axis. In the following we will generally refer to bias current and orthogonal magnetic field as *I* and *B*. The sensitivity *S_I_* depends on physical and geometrical parameters. It is usually expressed as:

(2) $$S_{I}=G\frac{r_{H}}{qnt_{eff}}=G\mu_{H}R_{sq}$$

where *n* is the concentration of quasi-free carriers (which is almost equal to the doping level *N_D_* in n-doped silicon), *t_eff_* is the effective thickness of the n-well, *r_H_* is the Hall factor, *q* is the electron charge, *G* is the geometrical correction factor, and $R_{sq}={\left(q\mu_{n}nt_{eff}\right)}^{-1}$ is the square resistance of the Hall sensor. The effective thickness *t_eff_* represents the actual thickness of the sensitive region of the n-well narrowed by the depletion region occurring at the pn-junction (Figure 1). The Hall factor *r_H_* expresses the ability of the material to generate the Hall voltage. It mainly relies on scattering effects and material anisotropy and can be combined with the electron mobility so as to get the Hall mobility to $\mu_{H}=\mu_{n}\cdot r_{H}$. In silicon, the Hall factor is around unity and hence the Hall mobility is equal to the electron mobility (*µ_H_ ≈ µ_n_)*. *G* is a correction factor introduced to take into account the efficiency of the specific shape of the Hall probe [25,26].

### 2.3. Numerical Model

Synopsys Sentaurus Device^®^ was used in this work as a physical simulator for the Hall probe. It embeds the common drift-diffusion transport model enhanced with magnetic field-dependent terms taking into account the Lorentz force. The general equation for the electron current density is:

(3) $$\vec{J_{n}}=\vec{J_{0}}+\mu_{n}\frac{1}{1+{\left(\mu_{H}B\right)}^{2}}\left[\mu_{H}\vec{B}\times \frac{\vec{J_{0}}}{\mu_{n}}+\mu_{H}\vec{B}\times \left(\mu_{H}\vec{B}\times \frac{\vec{J_{0}}}{\mu_{n}}\right)\right],$$

where $\vec{J_{0}}$ is the current vector due to the applied electric field only, *µ_H_* is the Hall mobility, *µ_n_* is the electron mobility, $\vec{B}$ is the magnetic field vector and *B* is the magnitude of this vector [7]. Equation (3) is a general formulation of carrier transport in the presence of magnetic field, solving the Lorentz equation for each carrier. For this reason, (3) has no validity limitations but numeric convergence is reliable only for *B* < 10 T [7].

Other than transport equation and Lorentz force, the simulation also takes into account mobility degradation due to doping concentration, temperature and carrier-carrier scattering, high field saturation, and Shockley-Read-Hall recombination. Synopsis Sentaurus Device^®^ has been already proven as a fair and reliable tool for Hall probe analysis [18,20].

## 3. Experimental Section

In this section, TCAD simulations will be used to: (i) understand the effects of design parameters on sensitivity, power consumption and bandwidth of a general Hall probe and (ii) define a set of design rules. This paper does not deal with the offset since electronic techniques can be used to effectively reduce it [27].

### 3.1. Current-Related Sensitivity

First of all, we study the effects of geometrical dimensions on current-related sensitivity *S_I_*. Simulations were done on the sensors described in Section 2.1, biased with a constant 500-µA current. The current-related sensitivity *S_I_* is related to geometrical dimensions by means of the geometrical correction factor *G* as shown in (2). This factor has been defined as the ratio between the sensitivity of a real Hall probe and that of an infinitely long Hall probe [19,26]:

(4) $$G=\frac{S_{I}}{{S_{I}|}_{L=\infty }}$$

Following this definition, *G* tends to be 1 when *L* = *∞*. The *G* factor can be more precisely expressed as a complex function of *W, L,* and Hall angle *θ_H_*. The following equation is valid only for rectangular probes with *L* > 0.85*W* and 0 ≤ *θ_H_* ≤ 0.45, which implies weak magnetic field regime [19]

(5) $$G=1-\frac{16}{\pi^{2}}e^{-\;\frac{\pi L}{2W}}\left(1-\frac{8}{9}e^{-\;\frac{\pi L}{2W}}\right)\left(1-\frac{\theta_{H}^{2}}{3}\right).$$

**Figure 2:**
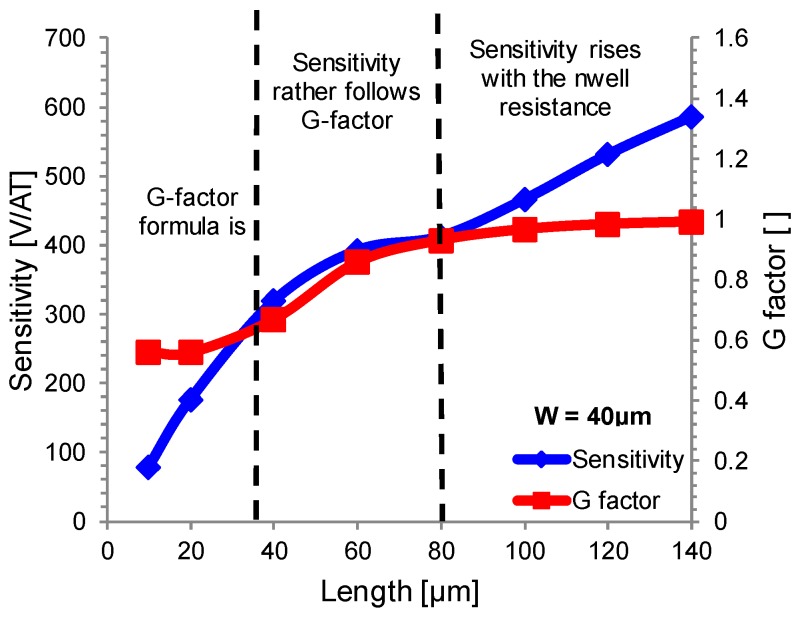
Current-related sensitivity *S_I_* at different sensor lengths simulated for a 40 µm wide sensor. Sensitivity follows the *G* factor only for a small range of lengths. Sensitivity keeps on rising for *L* > 80 µm due to the lowering of the effective thickness of the n-well.

The effect of the specific geometry on the *G* factor has been profusely studied in many articles [18,19,20,22], but the geometries indirectly affect also other parameters. Figure 2 compares (5) with the simulated current-related sensitivity for a Hall probe with constant bias current, *W* = 40 µm, and sweeping the length from 10 µm to 140 µm.

**Figure 3:**
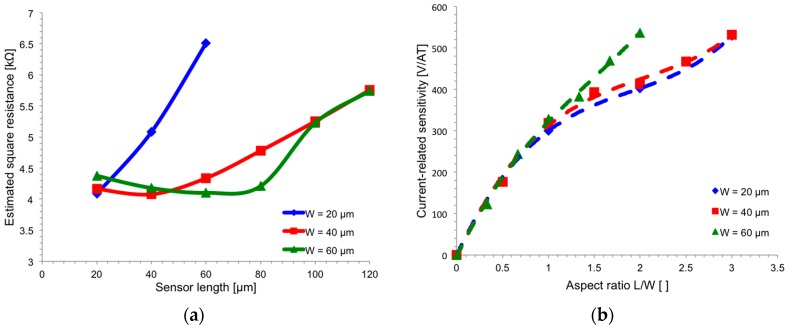
(**a**) Simulated square resistance as a function of the sensor length for different sensor widths. Higher voltage is needed to keep the same bias current in longer sensors, and this higher voltage implies an effective thickness lowering, resulting in increased square resistance; (**b**) Simulated current-related sensitivity *S_I_* as a function of geometrical aspect ratio *L*/*W* for different sensor widths. Lines are obtained by means of a third-order polynomial fitting.

Comparing both curves, it is possible to see that simulated *S_I_* fits the logarithmic behavior of *G* factor only for a small range of lengths. Sensitivity falls down very quickly for *L* < 0.8*W* = 34 µm. This behavior is not predicted by (5) since it is not valid in this region. On the other hand, sensitivity rises unbound as length increases, while (5) predicts the existence of an asymptotic value for *L*–>*∞*. This discrepancy clearly indicates that geometries alters other parameters than the *G* factor. A possible explanation is the increased resistivity of the n-well with the length of the sensor (Figure 3a). A longer sensor means higher resistance between bias contacts, thus higher bias voltage is needed to keep the same current, thereby changing the effective thickness of the n-well as it modulates the depletion region of the reversed biased pn-junction. As a result, a long n-well is preferable to obtain high current-related sensitivity. This simulation was done for three different sensor widths: 20 µm, 40 µm, and 60 µm, reporting similar results as shown in Figure 3b, where the sensitivity is reported with respect to the aspect ratio rather than the single geometrical dimensions. Sensor with *W* = 60 µm behaves in a slightly different way because its resistivity shows a steep increase for *L* = 100 µm (Figure 3a).

### 3.2. Power Consumption

The conclusion of the previous section is that the longer the n-well, the higher the sensitivity *S_I_*. However, for *L* ≥ 2*W,* the increase in sensitivity is mainly related to the reduction of the effective thickness of the n-well *t_eff_* (i.e., resistance growing) (Figure 3), leading to higher power consumption for a fixed bias current. The total resistance of the n-well is given by:

(6) $$R=R_{sq}\frac{L}{W}=\frac{1}{q\mu_{n}nt_{eff}}\frac{L}{W}$$

where *R_sq_* depends on the effective thickness of the n-well. Assuming a constant bias current *I*, the resistance *R* grows linearly with *L* when the applied voltage *V* is small (i.e., small values of *R* and *L*), while it grows more than linearly for higher values of the applied voltage, due to the widening of the depletion layer between n-well and p-substrate. On the contrary, the sensitivity *S_I_* has a pseudo-logarithmic behavior, as discussed in Section 3.1. This suggests the existence of an optimum point, which is a value of the aspect ratio that optimizes the trade-off between sensitivity and power.

For a better understanding it is useful to define an efficiency factor *η* expressing how much power is required to get a certain Hall voltage *V_H_* for a magnetic induction of 1 T:

(7) $$\eta =\frac{V_{H}}{V\cdot I\cdot B}=\frac{S_{I}}{V}\left[\frac{V}{W\cdot T}\right]$$

where *V* and *I* are the bias voltage and current, respectively. The *η* factor defines how much Hall voltage is generated when consuming 1 W at 1 T of magnetic induction. This parameter can be referred to as the power-related sensitivity of the sensor, since:

(8) $$V_{H}=\eta \cdot V\cdot I\cdot B=\eta \cdot P\cdot B$$

**Figure 4:**
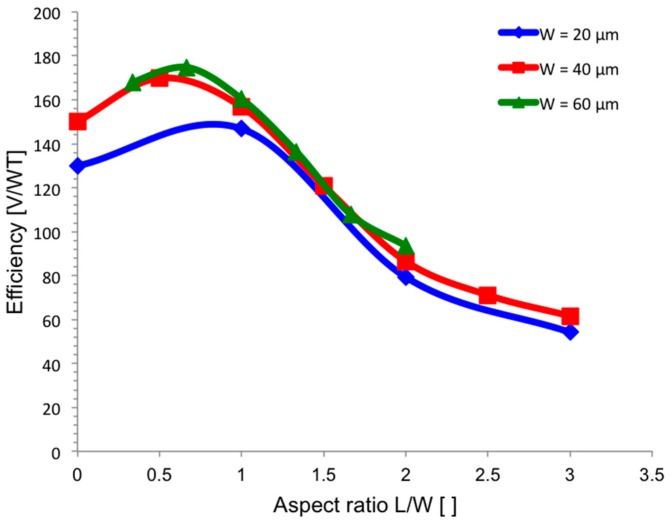
Simulated efficiency factor *η* for different aspect ratios *L*/*W* and different width *W* of the sensor. The bias current is kept constant at 500 µA. High efficiency is reached for square sensors.

Figure 4 reports the simulated efficiency factor *η* for Hall sensors with different aspect ratios *L/W* and same bias current *I* = 500 µA. A high-symmetry square-shaped sensor shows the best power efficiency, that is the best trade-off between current-related sensitivity and power consumption. In fact, long and narrow sensors provide higher sensitivity due to increased resistance of the n-well, as described in Section 3.1, but they also suffer from higher power consumption due to the same effect. Conversely, short and large sensors dissipate less power but the charge accumulation due to the Hall effect is negligible.

### 3.3. Optimum Square Dimension

**Figure 5:**
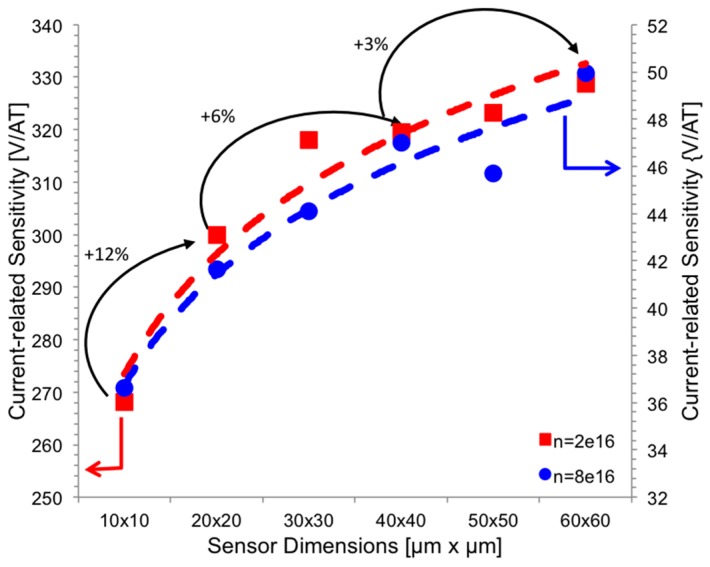
Current-related sensitivity *S_I_* simulated for square sensors with different areas and different doping concentrations (note two different scales were used on the y-axis. Left scale for the lowly doped sensor and right scale for the highly doped sensor). Small sensors suffer from boundary effects limiting sensitivity. Sensitivity rises as a logarithmic function of the area; hence a 30 µm × 30 µm is a good trade-off between area and sensitivity.

Once high-symmetry shapes are defined as the best choices, (1) and (2) state no other relations with the area of the sensor. However, there is another trade-off between area and performance to consider. Figure 5 shows the simulated *S_I_* for square Hall sensors with increasing areas. Small sensor suffers from boundary effects, limiting its sensitivity [25]; while large sensor, with an area above a certain value (let us say 30 µm × 30 µm), does not improve significantly the sensitivity, pointing out an asymptotic relation as the one shown in (5). Given the square sensor as the optimum geometry, a compromise between area and sensitivity exists and this relation does not depend on the doping concentration of the n-well. The blue line in Figure 5 shows the same simulation on a highly doped n-well ($N_{D}=8\;\times \;10^{16}\;{\mathrm{cm}}^{-3}$). Nominal sensitivity has fallen to values between 30 and 50 V/AT, but the behavior is still the same.

### 3.4. Dimension of Contacts 

**Figure 6:**
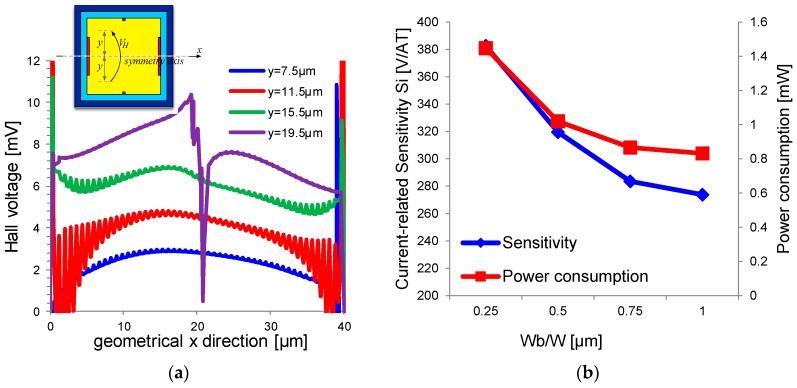
(**a**) Simulated Hall voltage along x direction for a square 40 µm × 40 µm sensor. Hall voltage is measured at four different distances from the symmetry axis. Hall voltage increases when moving away from the symmetry axis as the carriers’ path are more bent; (**b**) Simulations on a square 40 µm × 40 µm sensor with different width *W_B_* of bias contacts.

Sense contacts must be as small as possible, allowing more punctual and accurate measurements [19]. They must be placed at the center of the n-well, i.e., *x* = *L*/2, where the maximum of the Hall voltage is developed. They must also be placed close to the edges of the n-well, along the y-axis, since the Hall voltage grows as we go far from the center of the sensor, as shown in Figure 6a, which is the result of a simulation on a square 40 µm × 40 µm sensor. The figure shows the Hall voltage ideally measured at different points on the y-axis. The spike on the *y* = 19.5 µm curve at *x* ≈ 20 µm is due to the presence of the highly doped contact while the oscillations relate to numerical errors.

Dimensions of biasing contacts affect both the power consumption and the sensitivity *S_I_*, showing another trade-off. Reducing *W_b_* leads to higher contact-to-contact resistance, hence higher applied voltage is needed to keep the bias current constant. As said in previous sections, this leads to a reduction of the effective thickness of the n-well. Hence, small bias contacts are preferred in application requiring high sensitivity, while large bias contacts are preferred in application demanding for low power consumption. Bias contacts with *W_b_* = *W/2* are a good trade-off between power and sensitivity. Figure 6b shows the relation of *S_I_* and power consumption with the normalized dimension of *W_b_/W*.

### 3.5. Bias Current

**Figure 7:**
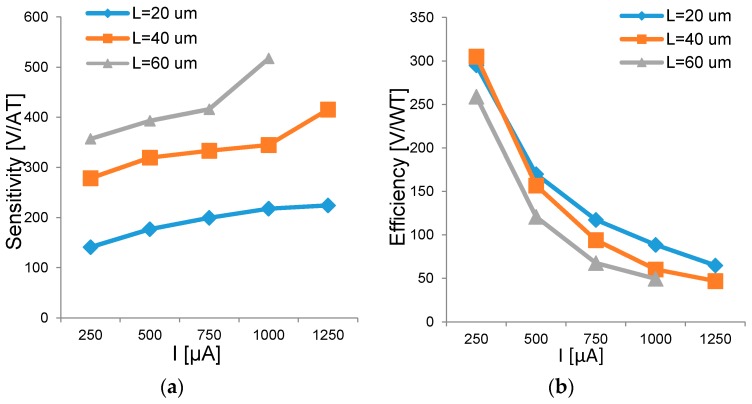
(**a**) Sensitivity and (**b**) efficiency versus bias current simulated for sensors with different aspect ratios but constant *W* = 40 µm.

Following (2), the current-related sensitivity *S_I_* is independent of the bias current. However, simulations show a small increase in sensitivity with higher bias currents (Figure 7a). Increasing the bias current has not direct effect on *S_I_* but lowers the effective thickness of the n-well, since higher bias voltage is applied. This is the same effect we observed and described in Section 3.1. Figure 7 reports both the current-related sensitivity *S_I_* and the efficiency factor *η* for sensors with different geometries and sweeping the bias current from 250 µA to 1.25 mA. High bias current leads to high power consumption, following *P = RI^2^*, and high sensitivity *S_I_* at the same time. Therefore, the bias current defines another sensitivity/power trade-off that must be properly adjusted based on the application constraints. These results are in agreement with the measurements and simulations carried on Hall probes with dissimilar shapes reported in [20].

### 3.6. Measurement Comparison

**Figure 8:**
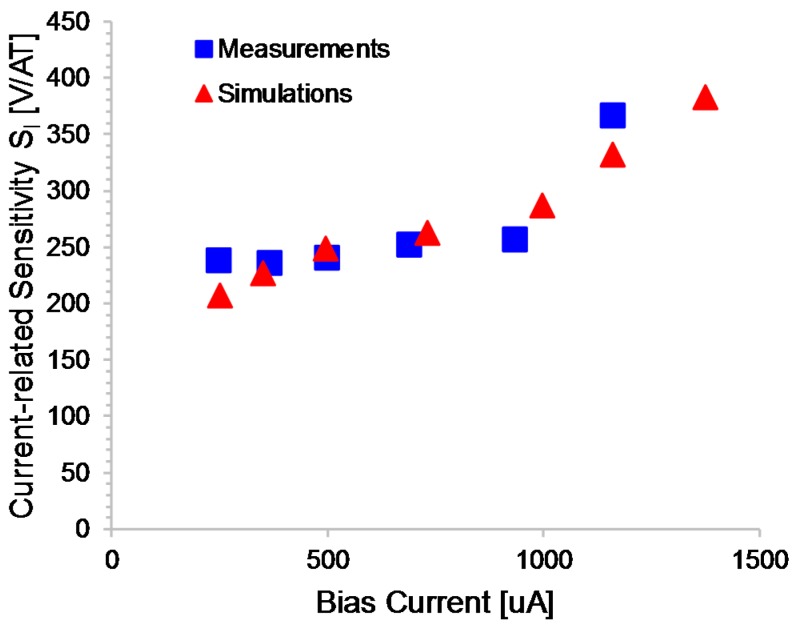
Comparison between measurements and simulations of the current-related sensitivity *S_I_* for a square-shaped Hall sensor with four equal contacts placed on the angles of the n-well. Both measurements and simulations reveal an increase of the sensitivity at high bias current that can be explained by the widening of the depletion layer between the sensitive n-well and the p-substrate. This result is in agreement with all the previous simulations.

Following the above-defined rules, a square-shaped 30 µm × 30 µm Hall sensor with four identical square contacts placed at the angles of the n-well was fabricated in CMOS 0.16 µm technology. Current-related sensitivity at various bias currents was measured and compared with TCAD simulations in Figure 8. The measurement proves the good accuracy of the simulations and demonstrates the narrowing of the effective thickness of the n-well since the increase of the sensitivity *S_I_* when applying *I* > 1 mA is evident.

### 3.7. Bandwidth

**Figure 9:**
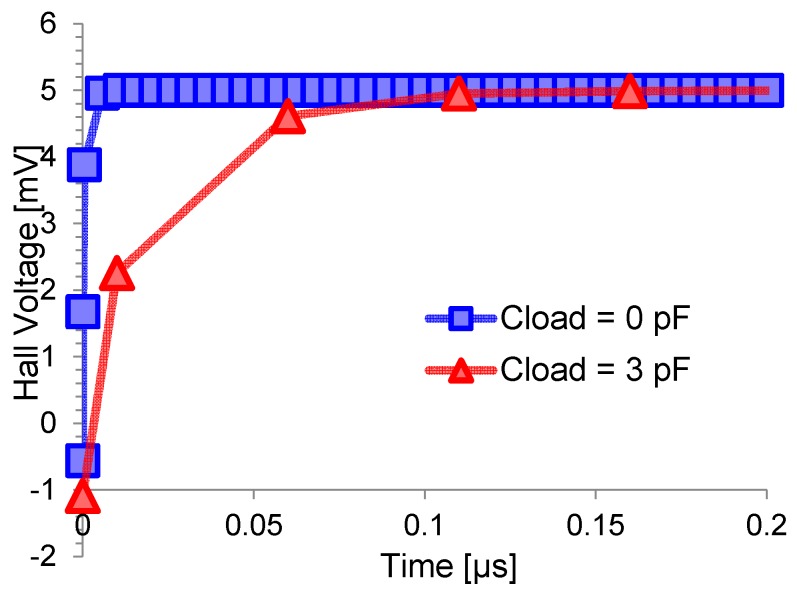
Hall voltage *V_H_* in response to a step change of 50 mT in the applied magnetic induction for a sensor with floating sense contacts (**blue line**) and with 1.5 pF capacitive load on each contact (**red line**).

**Figure 10:**
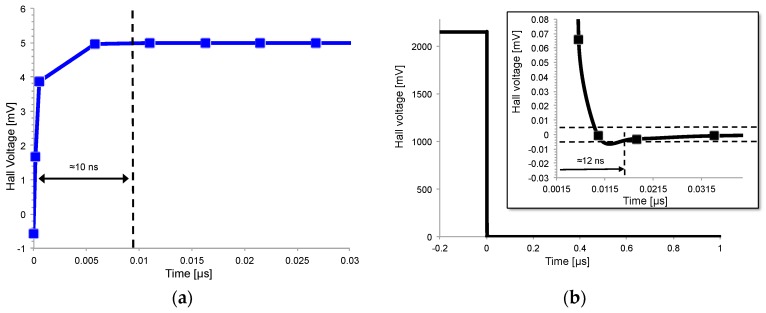
(**a**) Settling time of the Hall voltage after a 50 mT step change of the magnetic induction; (**b**) settling time of the Hall voltage after a step change of the bias current. Hall voltage is normalized to its steady state value. Transient ends when Hall voltage reaches its steady-state value within 0.1% error.

This section will analyze bandwidth and step response. We define the settling time in response to a step change time as the time to reach the steady-state voltage within an error lower than 0.1%. Figure 9 shows the Hall voltage *V_H_* in response to a step change of magnetic induction from 0 to 50 mT for two values of capacitive load at the sense contacts, which are floating contacts (blue line) and 1.5 pF-loaded contacts (for a total of 3 pF) (red line). The simulations were done on a square 30 µm × 30 µm sensor with four identical contacts of 1 µm × 4.5 µm placed at the angles of the n-well. The sensor responds faster with reduced capacitive loading, demonstrating the importance of the interface between the sensor and the electronic front-end. In fact, the upper bandwidth limit of Hall sensor is set by the capacitive load, as recently proven by the authors in [28,29]. The settling time can be modeled as an RC time constant where *R* is the resistance of the n-well and *C* is given by the capacitance of the n-well and the capacitive loads of the electronic front-end. This RC time-constant models the rearrangement of carriers inside the sensor in response to a stimulus. It enters into play regardless if the source of the stimulus is a step of the magnetic field or a step of the bias current, as shown in Figure 10, where the sensor (in this case without capacitive load) responds in about 10 ns to any kind of stimulus.

**Figure 11:**
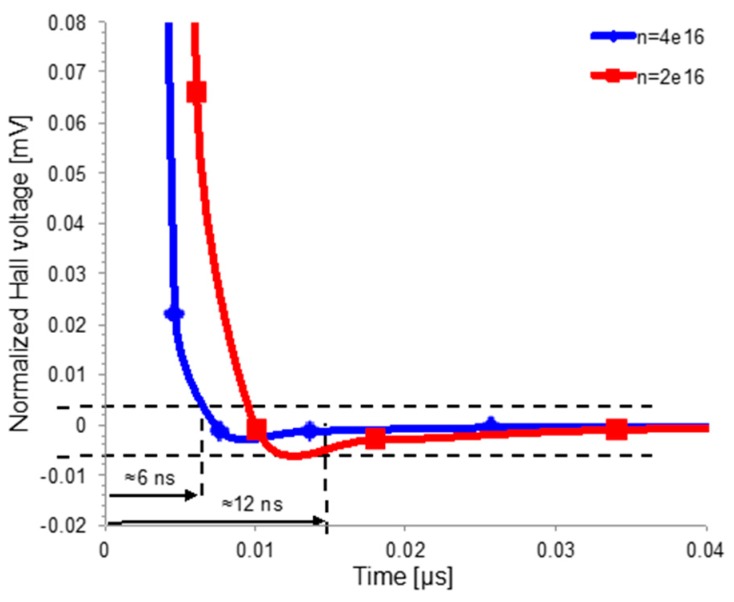
Effect of the n-well doping concentration (*N_D_ = n*) on the settling time. Doubling the doping concentration halves the response time. However, higher doping level lowers the sensitivity following (3).

To further prove the RC-like behavior, a step of the bias current was applied to two similar sensors differentiating each other only by the doping level of the n-well. Doubling the carrier concentration, *n* changes the well resistivity from 2.9 Ωcm to 1.7 Ωcm keeping the junction capacitance almost the same. As expected, Figure 11 shows a reduction of the response time by a half.

## 4. Discussion

Sensitivity is affected by the geometrical characteristics through the geometrical correction factor *G*, but it also depends on the effective thickness of the n-well, which is modulated by the bias voltage. For a fixed and constant bias current, a longer Hall probe requires higher bias voltage, thereby reducing the effective thickness of the n-well and increasing the probe resistivity. This relation causes a proportional increase of the sensitivity with the length of the sensor, even after the saturation of the *G* factor (Figure 2). This resistive-induced increase of the sensitivity is at the detriment of power consumption. The power efficiency factor, which can be referred to as a power-related sensitivity, points out the existence of an optimum aspect ratio which maximizes the sensitivity for a given power consumption (Figure 4). This optimum point is obtained for nearly-square, or high-symmetry, Hall probes. This is a very good result that allows the implementation of the current-spinning technique, which is the state-of-the-art procedure for offset reduction in Hall probes. The technique consists in spatially rotating the bias current by 90°, hence the sensor needs to be symmetric so as to correctly implement the current-spinning method [12,26,27].

Ohmic contacts influence sensitivity and power consumption, as well. To maximize the sensitivity, the sense contacts must be small, placed at the middle of the probe along the bias direction, and placed as close as possible to the edges of the probe along the orthogonal axis. Sense contacts have no effect on power consumption since no current flows through them (Figure 6), while bias contacts are prone to sensitivity/power trade-off. The implementation of the current-spinning technique imposes another trade-off on the contacts design: they must have the same shape since they swap continuously.

Also, the bias current is prone to a sensitivity/power trade-off, but it is more complicated to analyze since the bias current has a twofold effect: it changes the effective thickness and directly affects the Hall voltage *V_H_*, as shown in (1). As a result, the choice of the optimum value for the bias current is driven by the target application.

Finally, the dynamic behavior of the sensor can be accurately modeled as an RC circuit where *R* is the resistance of the sensor and *C* takes into consideration the well capacitance and all the capacitances connected to the sense contacts. From this model, wide acquisition bandwidth can be achieved by means of (i) minimized capacitive load at sensors contacts; (ii) the use of highly doped n-well as sensing probe. This second solution defines a sensitivity/bandwidth trade-off in Hall sensor design linked to sensor resistance:

(9) $$\begin{array}{l} S_{I}=G\mu_{H}R_{sq}\\ BW\approx \frac{1}{2\pi R_{sq}C} \end{array}$$

## 5. Conclusions

This manuscript has exploited modern technology-computer-aided-design (TCAD) simulations to investigate the effects of design parameters on sensor performance. Simulations are based on Synopsis Sentaurus^®^, which implements galvanomagnetic transport mode, and are verified through experimental measurements on a prototype. The analyses yield the following important trade-offs and design rules for CMOS Hall sensor design:

- Geometric characteristics of the probe affect the current-related sensitivity *S_I_* by means of both direct and indirect effects; which are associated with the geometrical correction factor *G* and the bias-induced change of the well resistivity, respectively.
- Square sensors, or at least symmetrical geometries, show the optimum sensitivity/power trade-off, i.e., the best energy efficiency. This is a good result since symmetrical sensors easily allow offset cancellation through spinning current technique.
- Optimum width of bias contacts is half the sensor width.
- Sense contacts should be as small as possible to have a punctual measure, hence maximize the sensitivity. They must be placed in the middle of the sensor along the bias axis (x-axis) and as far as possible from the center of the sensor along the orthogonal axis (y-axis).
- The intrinsic settling time of Hall sensor can be modeled as an RC time, where R is the resistivity of the n-well and C is the total capacitive effect acting on sense contacts. This is an important result since it reveals that the relatively slow responses of Hall sensor implementations are due to the readout architecture and not to physical constraints of the device. It also outlines an important sensitivity/bandwidth trade-off which is set by the resistivity of the n-well.
